# Bidirectional relationship between diabetes and pancreatic cancer: from mechanistic investigation to clinical application

**DOI:** 10.3389/fendo.2026.1995824

**Published:** 2026-10-22

**Authors:** Jiangmin Wu, Tingting Jiang, Xiaojia Li, Dacheng Xie, Keping Xie

**Affiliations:** 1 Center for Pancreatic Cancer Research, South China University of Technology, Guangzhou, China; 2 Department of Medical Oncology, Shanghai Pulmonary Hospital & Thoracic Cancer Institute, Tongji University School of Medicine, Shanghai, China

**Keywords:** antidiabetic agents, cancer prevention, diabetes, new-onset diabetes, pancreatic cancer, pancreatogenic diabetes

## Abstract

A well-established bidirectional causal relationship connects type 2 diabetes mellitus (T2DM) and pancreatic ductal adenocarcinoma (PDAC). Long-standing T2DM elevates pancreatic cancer risk approximately 1.8–2.2-fold, whereas 30%–74% of PDAC patients develop new-onset diabetes (NOD) 2–36 months before tumor detection, which mostly corresponds to tumour-originated type 3c pancreatogenic diabetes (T3cDM). Notably, 25%–40% of such patients achieve spontaneous glycemic recovery after R0 curative resection, solidifying the causal nature of PDAC-induced hyperglycemia. Six interrelated molecular mechanisms mediate their reciprocal crosstalk: chronic metabolic abnormality mediated by hyperinsulinemia and AGE-RAGE signaling, sustained inflammatory and macrophage remodeling, KRAS-driven oncogenic and epigenetic modification, pancreatic stellate cell-derived desmoplasia, tumor exosome-mediated β-cell damage, and gut-pancreas microbiome dysbiosis. Specifically, PDAC-secreted exosomes loaded with adrenomedullin, peritumor fibrotic islet destruction and circulating S100A8 peptide collectively lead to insulin deficiency, making T3cDM biochemically distinguishable from insulin-resistant classic T2DM by reduced pancreatic polypeptide and endogenous insulin concentrations. Clinically, END-PAC scoring and emerging machine learning algorithms facilitate risk stratification for early PDAC screening among patients aged 50 years or older with NOD. Nonetheless, prominent challenges remain: conflicting clinical data on the anti-PDAC potential of metformin and GLP-1RAs, ethnic heterogeneity limiting the generalizability of current prediction models, and up to 88% T3cDM misdiagnosis owing to the absence of global unified diagnostic benchmarks. Future translational research will center on prospective validation of multi-omic biomarkers, formulation of standardized T3cDM diagnostic criteria, large-scale screening trials, and rational repurposing of antidiabetic agents for pancreatic cancer prevention and adjuvant therapy.

## Graphical Abstract

**Figure:**
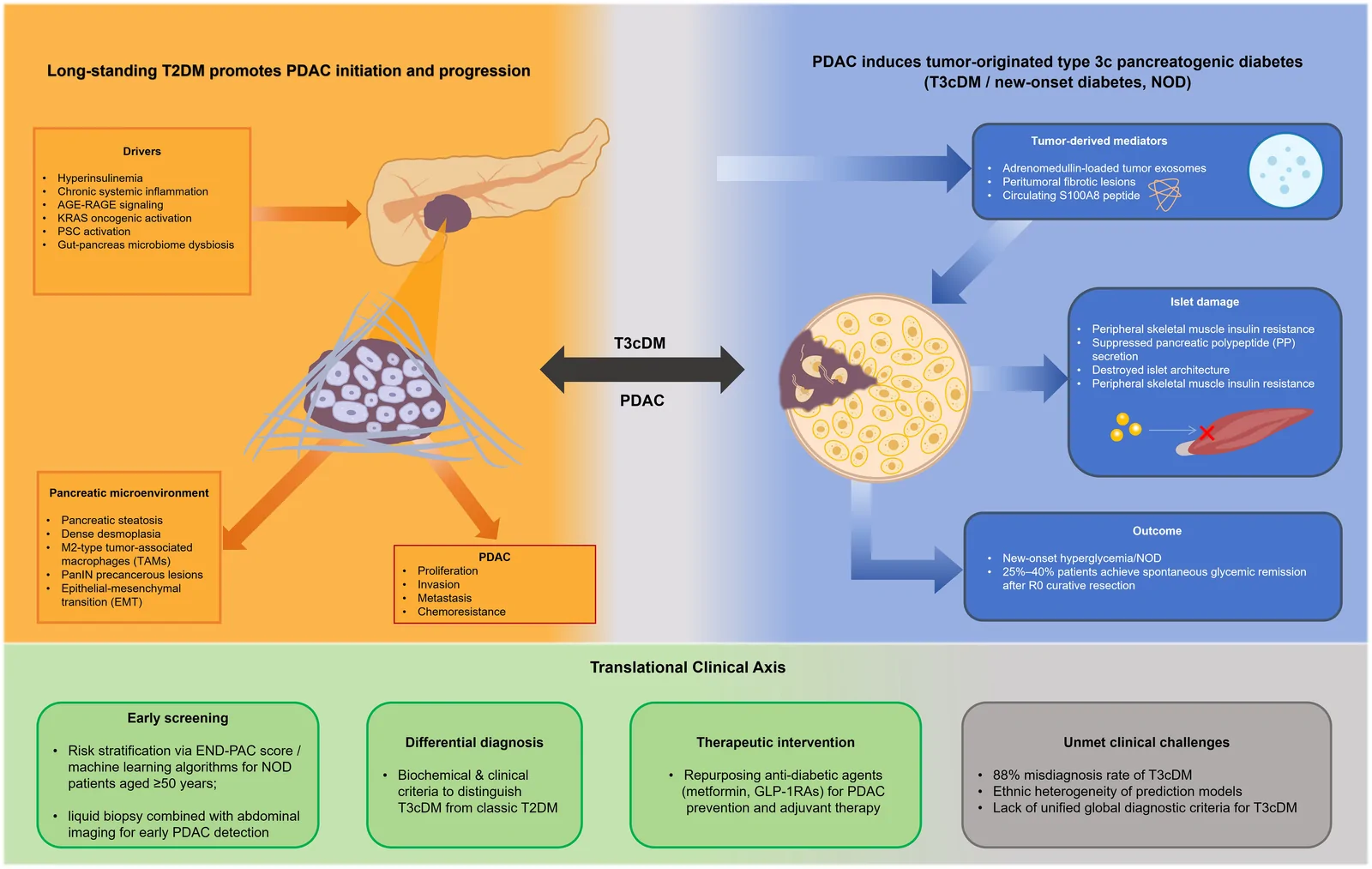
**Left Panel: Long-standing T2DM promotes PDAC initiation and progression.** *Upstream drivers*: Hyperinsulinemia, AGE-RAGE signaling, chronic systemic inflammation, KRAS oncogenic activation, pancreatic stellate cell (PSC) activation, gut-pancreas microbiome dysbiosis. *Intermediate pancreatic microenvironment*: Pancreatic steatosis, dense desmoplasia, M2-type tumor-associated macrophages (TAMs), PanIN precancerous lesions, epithelial-mesenchymal transition (EMT). *Downstream outcome*: PDAC proliferation, invasion, metastasis and chemoresistance. **Right Panel: PDAC induces tumor-originated type 3c pancreatogenic diabetes (T3cDM/NOD).** *Upstream tumor-derived mediators*: Adrenomedullin-loaded tumor exosomes, circulating S100A8 peptide, peritumoral fibrotic lesions. *Intermediate islet damage*: Pancreatic β-cell ER stress and apoptosis, destroyed islet architecture, peripheral skeletal muscle insulin resistance, suppressed pancreatic polypeptide (PP) secretion. *Downstream outcome*: New-onset hyperglycemia/NOD; 25%–40% patients achieve spontaneous glycemic remission after R0 curative resection. **Bottom Horizontal Translational Clinical Axis.** *Early screening*: Risk stratification via END-PAC score/machine learning algorithms for NOD patients aged ≥50 years; liquid biopsy combined with abdominal imaging for early PDAC detection. *Differential diagnosis*: Biochemical & clinical criteria to distinguish T3cDM from classic T2DM. *Therapeutic intervention*: Repurposing anti-diabetic agents (metformin, GLP-1RAs) for PDAC prevention and adjuvant therapy. *Unmet clinical challenges*: 88% misdiagnosis rate of T3cDM, ethnic heterogeneity of prediction models, lack of unified global diagnostic criteria for T3cDM.

## 1 Introduction

### 1.1 Epidemiological background

Pancreatic ductal adenocarcinoma (PDAC) remains one of the most lethal malignancies worldwide, with a 5-year survival rate of approximately 12% (1). The silent progression of PDAC, coupled with the lack of effective early detection strategies, results in most patients being diagnosed at an advanced, unresectable stage (1). In parallel, the global prevalence of diabetes mellitus (DM), particularly type 2 diabetes mellitus (T2DM), has reached pandemic proportions, affecting over 500 million adults.

The co-occurrence of DM and PDAC is strikingly high. Up to 45%–78% of PDAC patients exhibit new-onset or pre-existing abnormalities in glucose metabolism at the time of diagnosis (2). This clinical observation, first documented in the 19th century, has evolved over the past three decades from a mere statistical association into a well-recognized bidirectional causal relationship. Large-scale prospective cohorts, such as those from the Consortium for the Study of Chronic Pancreatitis, Diabetes, and Pancreatic Cancer (CPDPC) and the New-Onset Diabetes (NOD) study, have solidified the epidemiological foundation linking these two diseases (3, 4).

### 1.2 Key concept definitions

- Long-standing diabetes (LSDM): T2DM diagnosed over 3 years prior to PDAC detection (5). LSDM is considered an independent risk factor for PDAC development (6).
- New-onset diabetes (NOD): Diabetes mellitus newly diagnosed 2-36 months before PDAC diagnosis; in PDAC context, this represents a paraneoplastic phenomenon, a subtype of pancreatogenic diabetes (5).
- Type 3c pancreatogenic diabetes (T3cDM): Diabetes secondary to exocrine pancreatic disease (including chronic pancreatitis and PDAC), caused by combined β cell impairment, altered islet hormone secretion and pancreatic exocrine insufficiency (2, 7).
- PDAC associated diabetes: A subset of T3cDM caused specifically by pancreatic ductal adenocarcinoma (paraneoplastic new onset diabetes, NOD in PDAC patients) (2, 7).

Clinically, NOD presenting without elevated BMI or accompanied by unexplained rapid weight loss raises high suspicion for underlying PDAC related paraneoplastic T3cDM, in contrast to typical classic T2DM which is frequently associated with overweight/obesity.

### 1.3 Literature search strategy (PICO-framed)

- Population: Human subjects with type 2 diabetes mellitus or PDAC.
- Intervention/Exposure: Long-standing T2DM; PDAC-associated new-onset diabetes/T3cDM; anti-diabetic pharmacotherapies; risk-stratification screening models (END-PAC, machine-learning).
- Comparator: Non-diabetic subjects; classic T2DM patients without PDAC.
- Outcomes: Epidemiological risk estimates, molecular mechanistic evidence, diagnostic performance of biomarkers/models, and clinical outcomes (resectability, survival, glycemic remission after resection). We searched PubMed, Embase, and Web of Science using combinations of terms including diabetes, pancreatic ductal adenocarcinoma, new-onset diabetes, type 3c diabetes, pancreatogenic diabetes, exosome, screening, metformin, and GLP-1 receptor agonist. Searches were run up to March 2026. We prioritized meta-analyses, large prospective cohorts, mechanistic preclinical studies, and key consensus reports.

## 2 Epidemiological evidence for bidirectional association

### 2.1 Long-standing T2DM increases PDAC carcinogenic risk

A substantial body of meta-analytical evidence confirms that long-standing T2DM is an independent risk factor for PDAC. A comprehensive meta-analysis of 36 studies reported a pooled relative risk (RR) of 1.94 (95% CI: 1.66–2.27) for PDAC in patients with pre-existing T2DM (8). More recent updates, including studies through 2025, have maintained a consistent estimate of RR approximately 1.8–2.2 for T2DM patients compared to nondiabetic controls, as demonstrated by meta-analyses of 88 studies (RR = 1.97) (9) and of 23 studies with 30.9 million participants (HR = 2.39) (10).

The risk escalates with longer diabetes duration and poor glycemic control. Patients with T2DM lasting more than 5 years exhibit a significantly higher PDAC risk compared to those with shorter disease duration (5, 6). Importantly, Mendelian randomization studies have addressed the critical issue of reverse causality and residual confounding. Using genetic variants associated with T2DM as instrumental variables, these studies have demonstrated a causal effect of genetic predisposition to T2DM on PDAC risk, thereby ruling out the possibility that the association is merely due to early cancer-induced diabetes (2, 11).

Several high-risk subpopulations have been identified. Early-onset T2DM (diagnosed before age 55 years) carries a particularly high PDAC risk, with a standardized incidence ratio of 5.73 (95% CI: 4.49–7.22) (12). Additionally, T2DM complicated by obesity, nonalcoholic fatty liver disease (NAFLD), or pancreatic fat infiltration (lipomatosis) further amplifies PDAC risk through synergistic metabolic and inflammatory pathways (13).

### 2.2 PDAC induces type 3c pancreatogenic diabetes

The most compelling evidence that PDAC itself causes diabetes comes from epidemiological studies of NOD preceding PDAC diagnosis and, critically, from observations of postsurgical glycemic improvement. Between 30% and 74% of patients with PDAC are diagnosed with new-onset hyperglycemia or frank diabetes within 24–36 months preceding their cancer diagnosis (14). In prospective cohort studies, the incidence of PDAC among patients over age 50 with NOD is approximately 0.8%-1.0% within the first three years, representing a 6- to 8-fold increased risk compared to the general population (3).

A key causal proof is the observation of diabetes resolution following pancreatic tumor resection. Approximately 25%–40% of PDAC patients who undergo R0 surgical resection experience significant improvement or even complete normalization of their glycemic status postoperatively (15), indicating that the tumor itself was driving the diabetogenic state. Notably, this effect is most pronounced in patients with NOD, with resolution rates as high as 57% (6).

Type 3c pancreatogenic diabetes (T3cDM) secondary to PDAC is clinically and biochemically distinct from classical T2DM. Data from the CPDPC cohort have established that, compared to T2DM, PDAC-associated T3cDM is characterized by:

- Lower fasting and postprandial pancreatic polypeptide (PP) levels, reflecting damage to the pancreatic polypeptide-rich islets located in the pancreatic head (7);
- Lower adiponectin levels (16);
- Elevated IL-1Ra and other inflammatory markers (17);
- A predominant defect in insulin secretion rather than insulin resistance, accompanied by concurrent exocrine pancreatic insufficiency (17).

Key clinical-biochemical distinctions between PDAC-associated T3cDM and classic T2DM are summarized in **Table 1**.

**Table 1:** Distinguishing clinical and biochemical features between PDAC-associated T3cDM and classic T2DM.

Indicator	PDAC-related T3cDM (tumor-induced new-onset diabetes)	Classic type 2 diabetes mellitus (T2DM)
Baseline BMI & body weight	Normal/low BMI; rapid unexplained weight loss	Overweight or obese; weight loss uncommon without intentional dieting
Fasting insulin/C-peptide levels	Markedly decreased (primary insulin secretory defect)	Normal or elevated (dominant insulin resistance)
HOMA-IR (insulin resistance index)	Low	Significantly elevated
Pancreatic polypeptide (PP)	Greatly reduced	Normal or mildly elevated
Adiponectin	Elevated	Decreased
Pancreatic exocrine insufficiency (fecal elastase)	80% accompanied by exocrine dysfunction	Only ~30% show mild impairment
Inflammatory marker IL-1Ra	Elevated	Mildly elevated or within normal range
Glycemic change after radical tumor resection	25%–57% patients achieve spontaneous glycemic normalization	No improvement in glucose metabolism
Typical age of onset	Mostly middle-aged and elderly ≥50 years	Young adults to elderly population

Importantly, the onset of hyperglycemia and weight loss can precede radiographic evidence of PDAC by 12–36 months, providing a potentially critical window for early detection (4, 18).

### 2.3 Confounding factors affecting epidemiologic results

The interpretation of epidemiologic studies on DM-PDAC association requires careful consideration of several potential confounders. Obesity (BMI ≥30 kg/m²) is an independent risk factor for both T2DM and PDAC and may partially mediate the observed association (1, 19). Chronic pancreatitis, a well-established PDAC risk factor, frequently leads to T3cDM and can confound the DM-PDAC relationship if not properly excluded (17).

Lifestyle factors, including cigarette smoking and heavy alcohol consumption, increase the risk of both DM and PDAC and must be rigorously adjusted for in multivariate analyses (8). Certain medications, such as statins and low-dose aspirin, have been associated with modestly reduced PDAC risk in some large cohort studies and may represent important confounders (20). Finally, genetic susceptibility, including germline mutations in *BRCA1/2*, *CDKN2A*, *PALB2*, and other PDAC predisposition genes, also influences DM risk and requires careful handling in observational studies (21).

Key quantitative epidemiological evidence for this bidirectional causal relationship is compiled in **Table 2**.

**Table 2:** Core epidemiological evidence supporting bidirectional causal association between diabetes and PDAC.

Association direction	Core conclusion	Key quantitative data	Study types & cohorts
Long-standing T2DM increases PDAC risk	Chronic T2DM acts as an independent risk factor for PDAC development	Pooled relative risk (RR) = 1.8–2.2; risk rises further with diabetes duration >5 years; standardized incidence ratio = 5.73 for early-onset T2DM diagnosed before age 55	Meta-analysis of 88 cohort studies; Mendelian randomization studies
PDAC induces NOD	Pancreatic malignancy triggers paraneoplastic hyperglycemia classified as T3cDM	30%–74% PDAC patients develop NOD within 24–36 months before tumor detection; NOD patients ≥50 years have 6–8-fold higher 3-year PDAC risk than general population	Prospective NODES and CPDPC cohorts
Postoperative glycemic recovery verifies causality	Tumor resection reverses tumor-driven hyperglycemia	25%–40% patients gain significant glycemic improvement after R0 resection; remission rate up to 57% in NOD subgroups	Retrospective postoperative follow-up cohorts
Confounding modifying factors	Obesity, chronic pancreatitis, lifestyle and genetics interfere with DM-PDAC correlation	Obesity independently raises risks of both diseases; chronic pancreatitis frequently causes T3cDM; statin use slightly reduces PDAC risk	Large multivariate-adjusted population cohorts

## 3 Multidimensional mechanisms underlying bidirectional crosstalk

### 3.1 Mechanisms of T2DM promoting PDAC initiation and progression

#### 3.1.1 Metabolic dysregulation pathways (hyperinsulinemia/IGF-1 axis, AGE-RAGE, carbon stress)

Chronic hyperglycemia and hyperinsulinemia, hallmarks of T2DM, create a protumorigenic metabolic environment in the pancreas. Hyperinsulinemia, resulting from peripheral insulin resistance, directly stimulates pancreatic ductal epithelial cells via co-expression of insulin receptors (IR) and insulin-like growth factor-1 receptors (IGF-1R). Activation of the PI3K/AKT and MAPK signaling cascades promotes cellular proliferation, suppresses apoptosis, and enhances protein synthesis (2, 22).

Advanced glycation end-products (AGEs), which accumulate under chronic hyperglycemia, engage their receptor RAGE (receptor for advanced glycation end-products) on pancreatic epithelial cells. AGE-RAGE signaling activates NF-κB and ERK pathways, inducing epithelial-mesenchymal transition (EMT) and promoting progression from normal ducts to pancreatic intraepithelial neoplasia (PanIN) lesions (23).

Pancreatic fat infiltration (pancreatic steatosis) is increasingly recognized as a key driver in obese and diabetic patients. Ectopic fat deposition within the pancreas generates chronic lipotoxicity through free fatty acids and proinflammatory adipokines, creating a permissive microenvironment for malignant transformation (13).

#### 3.1.2 Chronic inflammation and immune remodeling (IL-1β+ macrophages, systemic low-grade inflammation)

T2DM is characterized by systemic low-grade inflammation, driven by metabolic stress in adipose tissue and the pancreas. Proinflammatory cytokines, particularly IL-1β, TNF-α, and IL-6, are elevated in the circulation and within the pancreatic microenvironment (7). These cytokines promote the recruitment and polarization of tumor-associated macrophages (TAMs) toward a protumorigenic M2 phenotype, which in turn enhances fibrosis, suppresses antitumor immunity, and facilitates PanIN progression to invasive PDAC (24).

Neutrophil extracellular traps (NETs) have recently emerged as critical mediators of DM-associated PDAC progression. Hyperglycemia-induced NETosis promotes tumor cell proliferation, metastasis, and thrombosis, representing a novel mechanistic link between diabetes and aggressive PDAC biology (25).

#### 3.1.3 Key oncogenes and epigenetic regulation (KRAS, YAP, Wnt/β-catenin)

The high-glucose microenvironment directly influences KRAS, the most frequently mutated oncogene in PDAC (>90% of cases). Hyperglycemia enhances KRAS stability and signaling output via post-translational modifications, while simultaneously activating the Wnt/β-catenin pathway, which synergizes with mutant KRAS to drive PDAC initiation (26–28).

Epigenetic reprogramming under diabetic conditions also contributes to PDAC susceptibility. Chronic hyperglycemia induces hypermethylation and silencing of the tumor suppressor lncRNA MEG3 (maternally expressed gene 3), while also modulating histone modifications at key cancer-related loci (29).

#### 3.1.4 Pancreatic stellate cell activation

Pancreatic stellate cells (PSCs) are the primary fibrogenic cells in the pancreas. Under normoglycemic conditions, PSCs remain quiescent. However, high glucose and AGE-RAGE signaling activate PSCs, converting them into cancer-associated fibroblasts (CAFs) (7, 30). Activated CAFs secrete excessive extracellular matrix components (collagen, fibronectin, hyaluronan), generating the dense desmoplastic stroma characteristic of PDAC. This fibrotic barrier not only promotes tumor progression but also creates a physical and biochemical shield that impedes drug delivery and confers chemoresistance (31, 32).

#### 3.1.5 Gut and pancreatic microbiota dysbiosis

Emerging evidence implicates the gut-pancreas axis in DM-associated pancreatic carcinogenesis. T2DM is associated with characteristic gut microbiome alterations, including reduced beneficial short-chain fatty acid-producing bacteria and increased abundance of proinflammatory taxa (33). Microbial metabolites, particularly lipopolysaccharide (LPS) and secondary bile acids, traverse the gut barrier, enter the portal circulation, and reach the pancreas, where they induce chronic low-grade inflammation and promote PanIN-to-PDAC progression in preclinical models (34).

The pancreatic microbiome, once considered sterile, is now recognized to harbor distinct bacterial signatures. Diabetic dysbiosis may further alter the intrapancreatic microbial community (35), with certain bacteria (e.g., Fusobacterium, Proteobacteria) enriched in PDAC tissues compared to normal pancreas (36).

### 3.2 Mechanisms of PDAC triggering type 3c pancreatogenic diabetes

While Module A addressed how T2DM promotes PDAC, this section focuses on the reverse direction: how PDAC, even at an early stage, induces new-onset type 3c diabetes (T3cDM). The key insight is that the tumor actively disrupts glucose homeostasis through secreted factors and local structural damage, often preceding radiographic detection by months to years.

#### 3.2.1 Tumor-derived exosome-mediated islet injury (core mechanism)

PDAC cells secrete diabetogenic factors that systemically impair pancreatic islet function. A landmark study by Javeed et al. demonstrated that exosomes-–nanoscale extracellular vesicles carrying proteins and nucleic acids-–are the predominant extracellular vesicles secreted by PDAC cells into culture media and patient plasma. These exosomes contain adrenomedullin and CA19-9, readily enter β-cells through caveolin-mediated endocytosis or macropinocytosis, and inhibit insulin secretion. Adrenomedullin receptor blockade abrogated the inhibitory effect of exosomes on insulin secretion. β-cells exposed to adrenomedullin or PDAC exosomes showed upregulation of ER stress genes and increased reactive oxygen/nitrogen species, indicating that PDAC causes paraneoplastic β-cell dysfunction through adrenomedullin-induced ER stress and failure of the unfolded protein response (37).

This exosome-mediated mechanism has been further characterized as an “exosomopathy,” where PDAC sheds adrenomedullin-containing exosomes into circulation that travel to β-cells and induce endoplasmic reticulum stress, leading to β-cell dysfunction and death. This knowledge could lead to improved diagnostic strategies for pancreatic cancer (18).

#### 3.2.2 Local destruction of islet architecture by tumor microenvironment

In addition to distant paracrine effects, the tumor physically and functionally disrupts islets within the pancreas. Using single-cell RNA sequencing and histopathological analysis, Hu et al. (2025) demonstrated that the PDAC tumor microenvironment is characterized by marked inflammation, severe fibrosis, and parenchymal atrophy. Tumor cells, immune cells, and fibroblasts interact with endocrine cells, activating apoptotic pathways in islet cells located proximal to the tumor (38).

Quantitative analysis revealed that islets in the proximal paratumor region exhibit significantly lower fractional β-cell area (0.68 ± 0.65% vs. 0.86 ± 1.02%, P = 0.037) and reduced islet density (0.54 ± 0.42 counts/mm² vs. 0.83 ± 0.90 counts/mm², P = 0.001) compared to islets in the distal paratumor region. This loss of β-cell mass is mediated by increased apoptosis, particularly in smaller islets, and is more pronounced in PDAC patients with T3cDM compared to those without diabetes (38).

Hill and colleagues further characterized these islet alterations through transcriptomic analysis of TCGA data. Histological examination revealed perivascular fibrosis and islet basement membrane thickening in PDAC patients, with the fibrotic reaction extending into the islets themselves. Transcriptomic data indicated significant downregulation of islet hormone genes insulin (INS) and glucagon (GCG) but not somatostatin (SST) in PDAC-associated diabetes, consistent with a T3cDM phenotype. Genes critical for β-cell function, including GCK (glucokinase), KCNK16 (potassium channel), DOC2A and RIMS2 (exocytosis regulators), as well as β-cell transcription factors MAFA and NEUROD1, were all downregulated in PDAC-associated diabetes. Importantly, no distinct immunogenic signature was identified, as key immune checkpoint genes (PDCD1/PD-1, CTLA4, CD274/PD-L1) did not show differential expression, suggesting that PDAC-associated diabetes arises primarily through neoplastic alterations in islet physiology rather than immune-mediated mechanisms (39).

Beyond direct islet parenchymal damage, PDAC driven fibro inflammatory remodeling also perturbs islet microcirculation. Inflammatory mediators and tumor secreted growth factors including TGF β and FGFs can disrupt vascular pericyte coverage, impair intra islet perfusion and disturb endocrine hormone release. Many of these growth factors are sequestered within the tumor stromal extracellular matrix; proteolytic matrix remodeling can liberate high local concentrations to act on adjacent islets. Apart from tumor derived exosomes, these soluble and matrix bound growth factors represent important paracrine mediators of islet dysfunction.

#### 3.2.3 Systemic insulin resistance induction via S100A8

PDAC-induced diabetes is not solely due to β-cell dysfunction; peripheral insulin resistance also plays a significant role. Basso et al. identified a 14-amino acid peptide (1589.88 Da) corresponding to the N-terminal of S100A8 that was present in pancreatic tumor samples from diabetic patients but absent in non-diabetic patients or non-neoplastic controls. At concentrations of 50 nmol/L and 2 mmol/L, this peptide significantly reduced glucose consumption and lactate production by cultured C2C12 myoblasts, caused lack of myotubular differentiation, and activated caspase-3. These findings indicate that the S100A8 N-terminal peptide impairs glucose catabolism by myoblasts *in vitro* and may cause hyperglycemia *in vivo* (40).

Subsequent work by the same group demonstrated that NT-S100A8 (500 nM) induced rapid insulin release and enhanced β-TC6 [Ca²^+^]i oscillations, while also activating Akt and NF-κB signaling in pancreatic cancer cells independently of insulin signaling. These observations suggest that S100A8-derived peptides exert pleiotropic effects on both β-cell function and peripheral insulin sensitivity (41).

#### 3.2.4 Transcriptomic and molecular evidence for β-cell dysfunction

Recent transcriptomic analyses have provided comprehensive molecular validation of the clinical observations. Hill et al. demonstrated significant downregulation of key islet hormone genes in PDAC patients with diabetes compared to those without diabetes. Specifically, INS (insulin) and GCG (glucagon) were significantly reduced, while SST (somatostatin) expression was preserved-–a pattern consistent with T3cDM rather than T2DM. Additionally, genes critical for β-cell function and insulin secretion, including the glucose sensor GCK (glucokinase), potassium channel KCNK16, exocytosis regulators DOC2A and RIMS2, and the β-cell transcription factors MAFA and NEUROD1, were all downregulated in PDAC-associated diabetes (39).

Importantly, no distinct immunogenic signature was identified in PDAC with diabetes, as key immune checkpoint genes (PDCD1/PD-1, CTLA4, CD274/PD-L1) did not show differential expression. This finding suggests that PDAC-associated diabetes arises primarily through neoplastic alterations in islet physiology rather than immune-mediated mechanisms, with implications for immunotherapeutic strategies (39).

### 3.3 Shared molecular pathways of bidirectional regulation

Despite the distinct initiating events (metabolic dysregulation in DM→PDAC vs. tumor-derived factors in PDAC→T3cDM), several intracellular signaling pathways serve as common hubs mediating bidirectional crosstalk. Key uncertainties requiring further investigation include the timing of systemic versus local drivers, the clinical relevance of tumor-derived signals in driving dysglycemia, the status of endocrine cell signaling and endocrine-exocrine crosstalk, and the effects of microvascular and matrix changes on endocrine-vascular exchange (39).

PI3K/AKT/mTOR Pathway: Hyperinsulinemia (in DM) activates PI3K/AKT in pancreatic epithelial cells, promoting proliferation and survival. Conversely, PDAC-derived factors (e.g., adrenomedullin, exosomal cargoes) activate stress pathways in islet cells, impairing insulin signaling and β-cell function via ER stress and unfolded protein response failure (18, 37).

TGF-β-Driven Fibrotic Pathway: A unifying mechanism emerging from recent literature is the role of pancreatic exocrine fibrosis as a common driver of islet dysfunction. The PDAC tumor microenvironment is characterized by marked inflammation, severe fibrosis, and atrophy, with activated pancreatic stellate cells and macrophages establishing a profibrotic signaling cycle. This pathway operates in both directions: DM-associated metabolic stress promotes PSC activation and fibrosis, while PDAC-induced desmoplasia similarly compromises islet function through fibrotic destruction (38).

ER Stress and Unfolded Protein Response: PDAC-derived exosomes deliver adrenomedullin to β-cells, inducing ER stress and failure of the unfolded protein response, leading to β-cell dysfunction and death. This mechanism represents a direct molecular link from tumor to endocrine dysfunction (37).

S100A8-Mediated Metabolic Disruption: The S100A8 N-terminal peptide represents a unique tumor-derived factor that both impairs peripheral glucose metabolism in myoblasts and alters β-cell calcium signaling, demonstrating how a single tumor product can exert multi-compartment effects on glucose homeostasis (40, 41).

In summary, these shared pathways operate as a two-way street: metabolic stressors in DM activate oncogenic signaling, while tumor-derived factors hijack the same pathways or induce parallel stress responses to disrupt glucose homeostasis, creating a self-reinforcing cycle that accelerates both tumor progression and metabolic deterioration (**Figure 1**).

**Figure 1:**
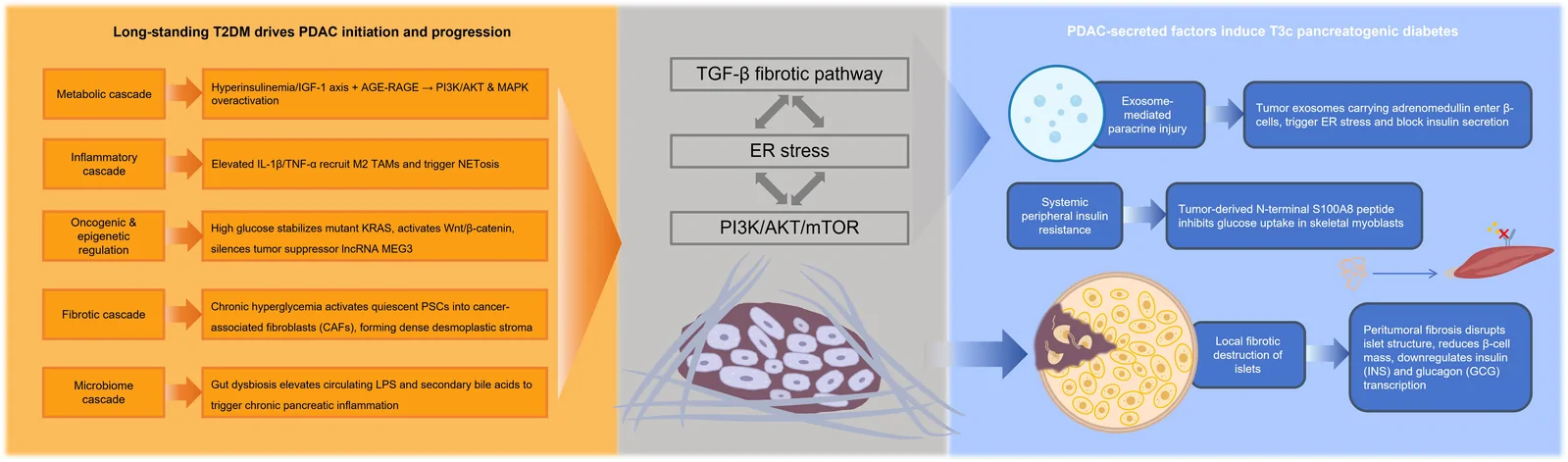
Schematic diagram of bidirectional molecular crosstalk between T2DM and PDAC. Color coding: Orange arrows represent tumor-promoting signaling cascades triggered by long-standing T2DM; blue arrows indicate diabetogenic factors secreted by PDAC that drive islet dysfunction; gray bidirectional arrows and gray boxes denote shared central signaling hubs involved in both pathological axes; black thin arrows denote downstream biological outcomes. Shape icons: Ellipses represent functional cell populations (pancreatic epithelial cells, β-cells, M2 tumor-associated macrophages, pancreatic stellate cells, cancer-associated fibroblasts); hollow spherical vesicles stand for adrenomedullin-containing tumor exosomes; rectangles correspond to metabolites, cytokines, protein mediators, oncogenes and tumor suppressors; irregular dark lesions represent pancreatic intraepithelial neoplasia (PanIN) and invasive PDAC; round clustered structures indicate pancreatic islets of Langerhans. The left panel illustrates five core pathways whereby chronic hyperglycemia and hyperinsulinemia facilitate PDAC initiation and progression. The right panel depicts three major mechanisms through which PDAC disrupts pancreatic endocrine function to induce type 3c pancreatogenic diabetes (T3cDM). The central zone highlights conserved overlapping signaling nodes, including the TGF-β fibrotic pathway, PI3K/AKT/mTOR cascade, and endoplasmic reticulum stress response, which mediate reciprocal crosstalk between T2DM and PDAC.

## 4 Clinical applications of DM-PDAC bidirectional relationship

### 4.1 NOD for early PDAC screening

#### 4.1.1 High-risk NOD population selection criteria

The recognition that NOD is associated with a 6- to 8-fold increased risk of PDAC within the first three years of diabetes diagnosis has established NOD as a critical window for early detection (3). Approximately 1% of individuals over age 50 with NOD will be diagnosed with PDAC within three years (3). However, screening all NOD patients is not cost-effective, necessitating risk stratification (42).

High-risk criteria (informed by Chinese 2021 pancreatic cancer screening consensus and END-PAC/EndPAC scores):

- Age ≥50 years with NOD (diagnosed within 24–36 months prior)
- Unexplained weight loss >5% of body weight
- Low or declining BMI (distinguishing from typical T2DM where obesity is common)
- Rapidly progressive or difficult-to-control hyperglycemia
- Family history of pancreatic cancer in first-degree relatives combined with NOD (strong screening indication)

Clinical prediction models: The END-PAC (Enriching New-Onset Diabetes for Pancreatic Cancer) score, derived from the Mayo Clinic cohort, assigns points based on three variables: age at diabetes onset, weight change, and change in blood glucose. A score ≥3 identifies high-risk individuals with a sensitivity of 78% and specificity of 82% (3). The model has been externally validated in multiple populations.

Recent prediction models incorporating machine learning and biochemical parameters have achieved AUCs up to 0.91 (43). However, challenges remain regarding missing data and cross-population validation (44).

#### 4.1.2 Screening pathway (imaging + biomarker stratification)

Risk-stratified approach: Using END-PAC criteria (score ≥3), approximately 20% of NOD patients are considered high-risk and warrant further investigation (**Figure 2**). A Markov model analysis by Vilalta et al. (2026) evaluated a cfDNA epigenomic blood test (Avantect) in high-risk NOD patients (END-PAC ≥3) (45). The cfDNA test has a sensitivity of 68% and specificity of 97%. The testing strategy demonstrated an incremental cost-effectiveness ratio (ICER) of $56,564 per quality-adjusted life year (QALY) gained, falling below the standard willingness-to-pay threshold of $100,000. In a modeled cohort of 10,000 NOD patients, the no-testing strategy identified 99 PDAC cases with only 7.1% resectable, whereas cfDNA testing identified 71 PDAC cases with 32.4% resectable, more than quadrupling surgical eligibility (45).

**Figure 2:**
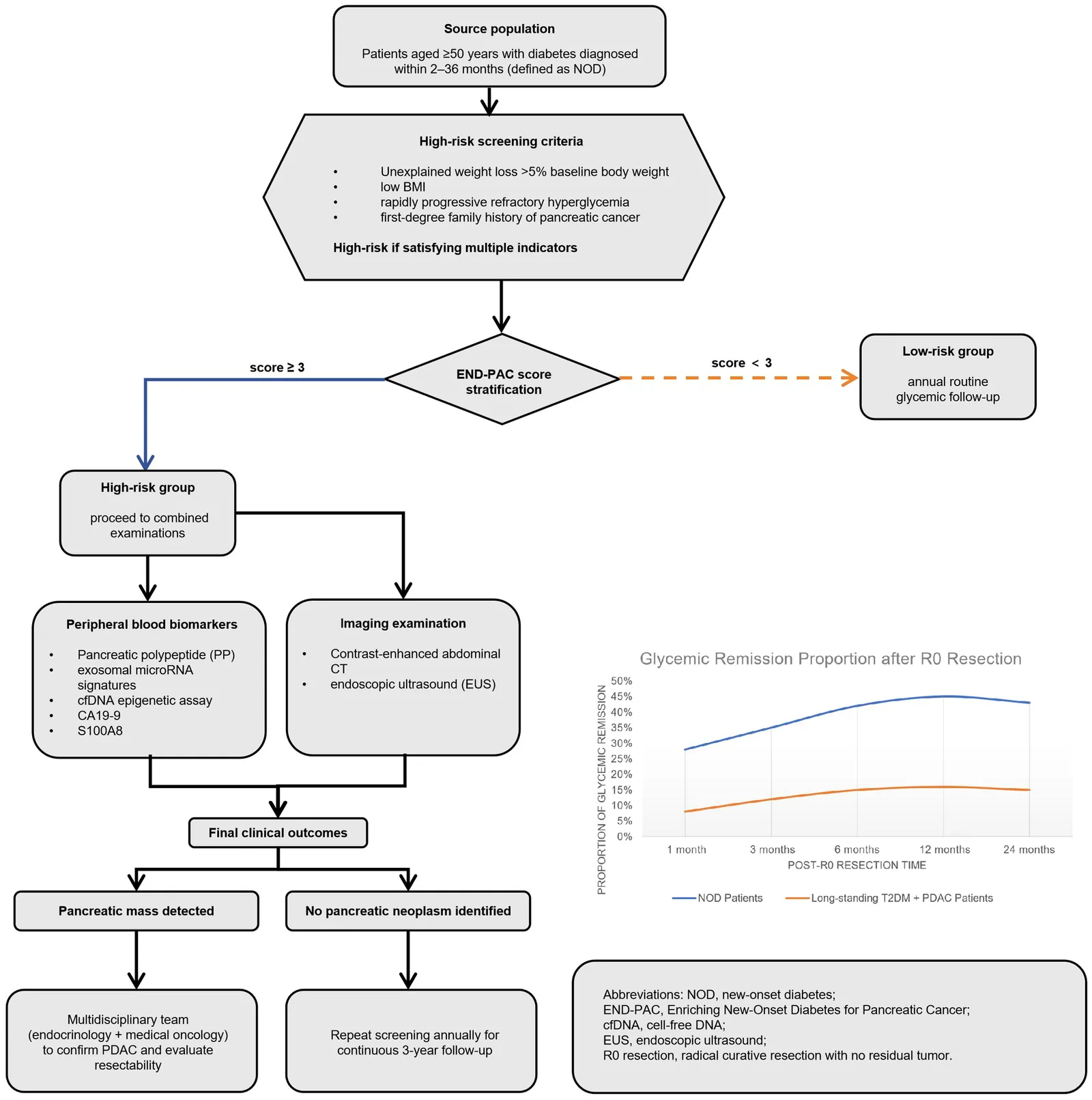
Clinical screening workflow for early PDAC risk stratification among patients with new-onset diabetes (NOD). Flowchart symbols: Rectangular boxes represent patient populations, clinical criteria, examinations and final clinical outcomes; diamond-shaped boxes denote risk judgment nodes based on high-risk indicators and END-PAC scoring cut-off value; solid black directional arrows show the sequential screening logic. The orange dashed branch indicates low-risk patients receiving routine annual glycemic follow-up; the blue solid branch marks high-risk individuals requiring combined biomarker detection and abdominal imaging assessment. Parallel testing modules include circulating biomarker panels (pancreatic polypeptide, exosomal microRNAs, cfDNA epigenetic assay, CA19-9, S100A8) and radiological examinations (contrast-enhanced abdominal CT, endoscopic ultrasound [EUS]). The inset line graph at the bottom right compares the proportion of patients achieving postoperative glycemic remission after R0 curative resection between NOD patients and patients with long-standing T2DM complicated by PDAC. All screened individuals without confirmed pancreatic lesions undergo continuous 3-year annual repeat screening. Abbreviations: NOD, new-onset diabetes; END-PAC, Enriching New-Onset Diabetes for Pancreatic Cancer; cfDNA, cell-free DNA; EUS, endoscopic ultrasound; R0 resection, radical curative resection with no residual tumor.

**Cost-effectiveness evidence:** An independent economic evaluation by the UK National Institute for Health and Care Excellence found that sequential use of a type 3c diabetes (T3cD) biomarker followed by a cancer-specific biomarker approached cost-effectiveness, with an ICER of £34,223 (US $45,809) per QALY (42). The analysis showed that 2.4 scans are required to detect one PDAC case among 200,000 people with NOD. Sensitivity analyses highlighted biomarker specificity as a critical determinant of cost-effectiveness (42).

Biomarker combinations for T3cDM vs T2DM distinction: Emerging multi-omic panels aim to distinguish PDAC-induced T3cDM from coincident T2DM:

- Pancreatic polypeptide (PP) - significantly reduced in PDAC-associated T3cDM (6)
- MicroRNAs, including exosomal miRNA signatures, show promise for early detection of beta-cell dysfunction in pancreatogenic diabetes (46, 47)
- CA19–9 shows improved utility when combined with other clinical parameters (44)

#### 4.1.3 Current limitations

- Screening models (END-PAC/EndPAC) were developed in predominantly Caucasian populations; Asian-specific calibration is needed.
- Single biomarkers lack sufficient sensitivity; multi-omics panels have not yet reached clinical implementation.
- Cost-effectiveness analysis suggests that screening becomes viable when 3-year cancer incidence exceeds 2% and 25% of cases are detected at a localized stage (44).
- Current detection rates fall below cost-effectiveness thresholds, highlighting the need for improved screening strategies (44).

Therefore, population level risk stratification for incident PDAC among patients with new onset diabetes would benefit from access to pre diagnosis longitudinal trajectories of HbA1c and body weight, referenced against age stratified population norms. However, HbA1c testing is not universally performed in primary care before diabetes/pre diabetes diagnosis. Enhanced longitudinal electronic health record documentation and heightened primary care awareness of abnormal metabolic trajectories (unexpected weight decline, abrupt glycemic rise without classic T2DM risk factors) may help enrich high risk individuals before formal diabetes diagnosis.

Risk-stratified imaging remains investigational rather than a universal routine clinical recommendation; further work-up may be considered only for high-risk individuals identified using validated scores such as END-PAC ≥ 3, while results from ongoing prospective trials including the EDI study are awaited. Repurposing metformin and GLP-1RAs for PDAC prevention or adjuvant therapy is pre-clinical and investigational, not standard of care, with observational associations distinguished from high-quality randomized trial evidence. No definitive clinical guidelines support universal routine pancreatic imaging for all patients with new-onset diabetes or repurposing glucose-lowering agents for pancreatic cancer prevention.

### 4.2 Antidiabetic drugs for PDAC prevention and prognosis modulation

#### 4.2.1 Metformin (most extensively studied)

Prevention: A randomized study from the Diabetes Prevention Program with 21 years of follow-up found that neither metformin (850 mg twice daily) nor intensive lifestyle intervention reduced cancer incidence among adults at high risk of diabetes. The hazard ratio for metformin compared with placebo was 0.90 (95% CI, 0.73-1.10) (48). The authors noted that these results may be impacted by increased nonstudy metformin usage over time due to the development of diabetes (48).

Prognosis in resected PDAC - Negative trial evidence: A multicenter randomized phase II trial (301P, ASCO 2025) evaluated adjuvant S-1 plus metformin versus S-1 alone in 76 radically resected PDAC patients [ASCO 2025, abstract 301P]. The trial found no significant improvement in 2-year overall survival (66.7% vs. 66.1%) or recurrence-free survival (17.9 vs. 12.6 months, HR 1.17, 95% CI 0.66-2.09, p=0.78) with metformin addition. The authors concluded that the addition of metformin at routine doses to resected PDAC patients receiving standard adjuvant chemotherapy did not significantly improve outcomes.

Mechanisms: Preclinical evidence indicates metformin inhibits mitochondrial complex I, reducing ATP and activating AMPK, suppresses CAF activation and desmoplasia, and enhances antitumor immunity.

Controversy: The lack of large-scale RCT evidence demonstrating benefit remains a limitation, and the DPP randomized data did not support a cancer-preventive effect of metformin in high-risk populations (21, 48).

#### 4.2.2 GLP-1 receptor agonists (semaglutide, liraglutide, etc.)

Pancreatic cancer risk - Meta-analysis evidence: A comprehensive systematic review and meta-analysis by Zou et al. (2026) including 60 studies with 4,932,290 patients (2,281,546 GLP-1 RA users) found that GLP-1 RA use was not associated with an increased PDAC risk overall (OR 0.688, 95% CI 0.575-0.824) (49). An inverse association was observed mainly in observational cohorts, in overweight/obese patients with diabetes, and with prolonged exposure (>52 weeks) and follow-up (>60 weeks) (49).

A separate meta-analysis by Chang et al. (2026) including 48 trials with 94,245 participants reported an odds ratio for pancreatic cancer of 0.84 (95% CI 0.53-1.35) with GLP-1RAs, indicating moderate-certainty evidence of no increased risk (49).

Conclusion: Current evidence does not support GLP-1RA use specifically for PDAC prevention, but provides strong reassurance for their use in diabetic patients at risk of or with PDAC. Repurposing for PDAC prevention or adjuvant therapy remains pre-clinical/investigational and is not a standard-of-care recommendation. Observational associations are clearly separated from high-quality randomized-trial evidence.

#### 4.2.3 Other antidiabetic agents

Insulin and sulfonylureas: Hyperinsulinemia has pro-proliferative effects on pancreatic epithelium. In high-risk or diagnosed PDAC patients, preference should be given to non-insulin agents when feasible (8).

#### 4.2.4 Statins and Aspirin

Large cohort studies suggest that long-term statin or low-dose aspirin use is associated with modest reductions in PDAC risk, likely through anti-inflammatory and anti-proliferative mechanisms (20).

### 4.3 Individualized management of PDAC complicated with diabetes

#### 4.3.1 Preoperative glycemic control

Distinguishing between coincident T2DM and PDAC-induced T3cDM is essential for perioperative management. T3cDM patients typically have lower BMI, lower fasting C-peptide and insulin levels (indicating insulin deficiency rather than resistance), and are more sensitive to exogenous insulin, putting them at higher risk of hypoglycemia if treated with standard T2DM insulin protocols (7).

#### 4.3.2 Systemic chemotherapy

Impact of DM on chemotherapy outcomes: The PANCAKE study (Mazzaferro et al., 2026), an Italian multicenter observational study of 663 advanced PDAC patients, found that pre-existing DM was associated with a modest but significant overall survival benefit (median 11.6 vs. 10.3 months, adjusted HR 0.82, 95% CI 0.67-0.99, p=0.036) (50). Importantly, this benefit was observed only in patients treated with first-line gemcitabine-nab-paclitaxel, not with FOLFIRINOX, suggesting regimen-dependent effects (50).

A longitudinal Bayesian joint modeling approach showed that a 10 mg/dL increase from baseline blood glucose was associated with a 4% reduction in the risk of death (HR 0.9967, 95% CI 0.9939-0.9995), indicating a statistically significant but clinically modest prognostic effect of glycemic trends during chemotherapy (50).

Clinical implications: Optimizing glucose control during chemotherapy may improve treatment tolerance and efficacy. Coordination between oncologists and endocrinologists is recommended.

#### 4.3.3 Postoperative management

Post-pancreatectomy diabetes: Total pancreatectomy results in T3cDM in a high proportion of patients, while partial pancreatectomy leads to T3cDM in approximately 20% of cases. Management typically requires basal-bolus insulin regimen, pancreatic enzyme replacement therapy (due to exocrine insufficiency), and close glucose monitoring.

Prognostic significance of postoperative glycemic improvement: Approximately 25%–40% of PDAC patients experience spontaneous glycemic improvement following R0 resection, with the highest rates observed in patients with new-onset diabetes (6, 15), which correlates with better overall prognosis (6).

#### 4.3.4 Prognostic differences between NOD-type and long-standing DM-PDAC

Some studies suggest that patients with NOD-type PDAC (tumor-induced T3cDM) may have different prognosis than those with long-standing T2DM preceding PDAC, possibly due to earlier diagnosis (the NOD itself prompting medical attention) or distinct tumor biology (2).

### 4.4 Differentiating type 3c DM from primary T2DM

Clinical challenge: T3cDM is frequently misdiagnosed as T2DM due to insufficient awareness among clinicians, leading to delayed pancreatic cancer screening and inappropriate diabetes management (7). Up to 80% of T3cDM patients may be initially misdiagnosed as T2DM, leading to delayed pancreatic imaging and cancer diagnosis (**Table 3**, *adapted from Bhattamisra* et al.*, 2019* (7)).

**Table 3:** T3cDM vs. T2DM: key distinguishing features.

Parameter	Type 3c DM (PDAC-associated)	Type 2 DM
BMI	Normal to low	Overweight/obese
Weight loss	Common, often unexplained	Uncommon or intentional
Fasting C-peptide	Low	Normal/high
Fasting insulin	Low	Normal/high
Insulin resistance (HOMA-IR)	Low	High
Pancreatic polypeptide	Markedly reduced	Normal or elevated
Exocrine dysfunction	Present (low fecal elastase)	Absent

A defining feature of T3cDM is its strong association with pancreatic exocrine insufficiency (PEI). PEI is much more common in T3cDM (80%) than in T1DM (20%) or T2DM (30%), indicating its diagnostic value (51).

Diagnostic approach - Triple modality: (a) *clinical* - Age at diabetes onset (≥50 years), absence of obesity, unexplained weight loss, rapid glycemic progression; (b) *biochemical* - Low C-peptide/insulin despite hyperglycemia (indicating insulin deficiency), low pancreatic polypeptide, elevated adiponectin; and (c) *imaging* - Abdominal CT or EUS to evaluate pancreatic morphology (tumor, chronic pancreatitis changes, atrophy, calcifications).

According to Ewald and Bretzel criteria, a diagnosis of T3cDM requires: (a) existence of pancreatic exocrine disease (51), (b) abnormal glucose metabolism consistent with diabetes, (c) absence of autoimmune markers typical of T1DM, and (d) objective evidence of pancreatic exocrine dysfunction (51).

Clinical consequence of misdiagnosis: Misdiagnosis leads to delayed pancreatic imaging and cancer diagnosis, inappropriate treatment with insulin sensitizers (which are ineffective for insulin deficiency), and failure to recognize and treat exocrine insufficiency (7).

Routine screening for pancreatic pathology should be considered in all patients over age 50 with new-onset diabetes who lack typical T2DM features (obesity, metabolic syndrome).

## 5 Current challenges and controversies

Despite substantial progress in understanding the bidirectional relationship between diabetes and pancreatic cancer, several critical challenges and controversies persist across mechanistic, clinical, and translational domains. This section synthesizes the most pressing unresolved issues that hinder the translation of research findings into clinical practice (**Table 4**).

**Table 4:** Summary of key controversies.

Domain	Challenge	Clinical Impact
Mechanism	Incomplete understanding of PDAC-DM pathophysiology	Delayed therapeutic targeting
Screening	3-year cancer incidence (0.8%) below cost-effectiveness threshold (2%)	Screening not routinely feasible
Models	END-PAC shows variable performance across ethnicities; limited external validation	Uncertain global applicability
Diagnosis	No standardized diagnostic criteria for T3cDM	~88% misdiagnosed as T2D
Drugs	Metformin evidence plagued by severe bias and low quality; GLP-1 RA safety concerns unresolved	Unclear risk-benefit profile
PEI	Exocrine insufficiency (80% in T3cDM) often overlooked	Incomplete management

### 5.1 Mechanistic gaps: incomplete understanding of causal pathways

Uncertainty regarding the timing and mechanisms of PDAC-associated diabetes. The pathophysiology of PDAC-associated diabetes is complex and incompletely defined. Key uncertainties include whether the primary driver is a tumor-derived systemic factor, local pancreatic microenvironmental changes disrupting islet architecture, or a combination of both. The status of endocrine cell signaling and endocrine-exocrine crosstalk in PDAC-DM remains poorly characterized (39). Additionally, the role of islet microcirculation, pericyte dysfunction, matrix-sequestered growth factors (TGF-β, FGFs) and matrix proteolytic remodeling in PDAC-associated T3cDM is also under-recognized.

Need for human *in vivo* validation of exosome-mediated mechanisms. While preclinical studies have established that pancreatic tumor-derived exosomes containing adrenomedullin can enter β-cells and impair insulin secretion through ER stress and unfolded protein response failure (37), the majority of these mechanistic insights come from *in vitro* systems and animal models. There remains a critical need for human *in vivo* validation of these pathways in PDAC patients.

Incomplete characterization of islet pathophysiology in PDAC-DM. PDAC-DM has distinct metabolic characteristics compared to type 2 diabetes. Unlike T2D patients who present with marked insulin resistance, patients with PDAC-DM present with comparatively greater insulin sensitivity, while beta-cell function is significantly more impaired. Additionally, alpha-cell dysregulation is present in PDAC, with an abnormally elevated postprandial glucagon response. Whether this dysregulation is a secondary phenomenon to insulin deficiency or represents an independent pathophysiological process remains uncertain (2).

### 5.2 Screening bottlenecks: from models to clinical implementation

*Insufficient cost-effectiveness of current screening strategies*. For screening to be cost-effectiveness modelling suggests that screening becomes viable when 3-year cancer incidence exceeds 2% and a sufficient proportion of cases are detected at a localized stage, whereas current detection rates (approximately 0.8%) and resectability rates (approximately 16%) fall below these benchmarks (3).

*Performance limitations of existing prediction models*. The END-PAC score exhibits reduced real-world performance and limited generalizability across non-Caucasian populations; strict inclusion criteria for new-onset diabetes further narrow its practical applicability (3, 52).

*Racial and ethnic heterogeneity*. The variable performance of prediction models across different ethnic groups represents a major barrier to global implementation and necessitates population-specific calibration and validation.

*Lack of validated biomarkers for routine clinical use*. Although numerous biomarkers have been proposed to distinguish PDAC-associated diabetes from T2DM, including adiponectin and IL-1Ra combinations achieving AUCs of 0.91 in research settings, none have undergone rigorous prospective validation in large, multi-center cohorts. This absence of validated biomarkers leads to substantial underdiagnosis of type 3c diabetes (2).

### 5.3 Pharmacological controversies: evidence gaps in drug repurposing

Metformin: severe bias and low-quality evidence. Metformin-associated PDAC benefits are undermined by major methodological limitations across retrospective studies. An umbrella review of 11 meta-analyses describing 24 individual studies found that all meta-analyses were rated as low or critically low quality (21). Most meta-analyses combined results from clinical trials and retrospective studies with different patient populations and control group definitions. Importantly, immortal time bias was present and not accounted for in 65.2% of the individual retrospective studies, and almost all meta-analyses failed to identify and correct for this source of bias. The authors concluded that meta-analyses describing the association of metformin use in patients with pancreatic cancer are plagued by various types of bias inherent in retrospective studies, and the quality of evidence linking metformin to decreased pancreatic cancer mortality is generally low (21).

GLP-1 receptor agonists: unresolved safety concerns. A meta-analysis of 62 RCTs comprising 66,232 patients found a significantly increased risk of pancreatitis (RR: 1.44, 95% CI 1.09-1.89, p=0.009), though this was not significant when stratified by background medication use. For pancreatic cancer, the overall risk showed no significant association (RR: 1.30, 95% CI 0.86-1.97). However, a significant increase was found in the subgroup with background medications (RR: 1.85, 95% CI 1.05-3.26, p=0.03), but not without background medications (RR: 0.81, 95% CI 0.43-1.55). This uncertainty represents an ongoing challenge for clinicians managing diabetic patients at risk of PDAC (53).

Insufficient evidence for most antidiabetic drug classes. The influence of anti-diabetic medications on pancreatic cancer development remains controversial. Although some evidence suggests that insulin may elevate cancer risk and metformin may reduce it, current findings have not reached a clear consensus (54).

### 5.4 Diagnostic confusion: widespread misclassification of type 3c diabetes

Substantial underdiagnosis and misclassification. T3cDM remains substantially under-recognized in clinical practice, with frequent misclassification as classic T2DM (7).

Lack of standardized diagnostic criteria. There are no standardized diagnostic criteria for T3cDM globally, unlike those for T1DM and T2DM. The best-known model is the criteria offered by Ewald and Bretzel, which require: (a) existence of pancreatic exocrine disease, (b) abnormal glucose metabolism consistent with diabetes, (c) absence of autoimmune markers typical of T1DM, and (d) objective evidence of pancreatic exocrine dysfunction. However, these criteria are not consistently applied in clinical practice (7).

Association with exocrine pancreatic insufficiency (PEI). A defining feature of T3cDM is its strong association with PEI. PEI is much more common in T3cDM (80%) than in T1DM (20%) or T2DM (30%), indicating its diagnostic value. However, this key distinguishing feature is often overlooked in clinical practice (7).

Overlap with type 2 diabetes features. PDAC-associated diabetes can resemble recent-onset type 2 diabetes early on, making it difficult to distinguish. PDAC-DM may present before frank exocrine insufficiency and then evolve as the tumor/stromal/islet pathology accrues, further complicating early diagnosis (2).

### 5.5 Clinical trial and evidence gaps

Absence of large-scale RCTs for screening strategies. Despite the promising potential of NOD-based screening for early PDAC detection, no large-scale randomized controlled trial has demonstrated that such screening improves overall or disease-specific mortality. The evidence base remains limited to observational studies, cost-effectiveness modelling, and small validation cohorts. The Early Detection Initiative (EDI) is currently addressing this gap (55).

Limited prospective validation of biomarkers. While the DM-PDAC Working Group within the CPDPC has established a biobank of clinically annotated biospecimens from over 2,270 new-onset diabetes subjects and is conducting Phase 3 validation studies of promising biomarkers, results are still forthcoming (56).

Lack of integration between oncology and diabetology. Few clinicians specialize at the intersection of oncology and diabetology. In day-to-day practice, the lethality of PDAC often pushes hyperglycemia from type 3c diabetes to the background, perpetuating the cycle of underdiagnosis and delayed detection.

Need for consensus classification criteria. Consensus classification criteria for T3cDM are needed to facilitate consistency in study design for future epidemiological and translational studies (7).

## 6 Future research directions

Building upon the current challenges and controversies outlined in *Section* 5, this section delineates priority areas for future investigation across basic, translational, and clinical domains. The overarching goal is to transform the bidirectional relationship between diabetes and pancreatic cancer from a mechanistic observation into actionable clinical strategies for early detection, prevention, and improved patient outcomes (**Table 5**).

**Table 5:** Summary of future research priorities.

Domain	Priority	Key Studies/Resources
Basic mechanisms	Elucidate tumor-derived signals causing β-cell dysfunction	Hill et al., 2025 (39); CPDPC DETECT study (56)
Biomarkers	Validate multi-omic panels for early detection	PaCANOD (59); CPDPC biobank (56)
Prediction models	External validate and improve ML models	XGBoost (44); Chen et al., 2023 (43)
Clinical trials	Complete EDI and conduct mortality trials	EDI RCT (55)
Drug repurposing	Identify optimal GLDs for PDAC prevention/treatment	Luconi et al., 2025 (54)
Clinical guidelines	Establish T3cDM diagnostic criteria	CPDPC consensus development (56)
Shared biology	Characterize common genomic biomarkers	Alam et al., 2025 (57); Poursheikhani et al., 2025 (58)

### 6.1 Basic and translational research priorities

#### 6.1.1 Elucidating the pathophysiology of PDAC-associated diabetes

As highlighted by Hill et al. (2025) and the CPDPC consortium, critical unresolved mechanistic questions persist, including the relative contribution of systemic tumor-derived signals versus local microenvironmental injury, endocrine-exocrine crosstalk, and microvascular-matrix remodeling to PDAC-associated islet dysfunction (39, 56).

Priority research questions include:

- What are the specific tumor-derived factors (exosomal cargo, cytokines, metabolites) that mediate β-cell dysfunction?
- How do the pancreatic tumor microenvironment and fibrotic changes impact islet architecture and function?
- What is the temporal relationship between PDAC development and the onset of dysglycemia?
- Can reversal of PDAC-associated diabetes following tumor resection be used as a biomarker of treatment efficacy?
- What is the role of islet microcirculation, pericyte dysfunction, matrix sequestered growth factors and matrix proteolytic remodeling in PDAC associated T3cDM?

#### 6.1.2 Multi-omics and biomarker discovery

The application of multi-omics technologies offers unprecedented opportunities to dissect the bidirectional relationship between diabetes and PDAC.

Genomics and transcriptomics. Recent bioinformatics analyses have identified shared genomic biomarkers between type 2 diabetes and pancreatic cancer. Using weighted gene co-expression network analysis (WGCNA) and protein-protein interaction network analysis, Alam et al. (2025) identified six top-ranked common differentially expressed genes (TOP2A, BIRC5, RRM2, ALB, MUC1, and E2F7) as common genomic biomarkers linking T2DM and PDAC (57). Future studies should validate these biomarkers in prospective cohorts.

Additionally, a study by Poursheikhani et al. (2025) identified that CEBPZ gene expression was significantly increased in blood samples of diabetic, pancreatic cancer, and pancreatic cancer-diabetic groups compared to controls, suggesting that CEBPZ expression may serve as a diagnostic biomarker for assessing PDAC in individuals with T2DM (58).

Proteomics and metabolomics. The CPDPC’s DETECT study, which uses a mixed meal tolerance test (MMTT) with a standardized collection protocol, is generating preliminary data on metabolic distinctions between CP-DM, PDAC-DM, and T2DM controls (56). Future research should expand these findings to develop validated diagnostic panels.

MicroRNAs and non-coding RNAs. Biomarkers such as microRNAs offer potential as dual sentinels for diagnosing and prognosticating both diabetes and cancer (46). Future studies should focus on validating specific miRNA signatures in large, prospective cohorts.

#### 6.1.3 Drug repurposing and novel therapeutic targets

Metformin and other glucose-lowering drugs. The multifaceted actions of glucose-lowering drugs (GLDs) in cancer treatment warrant further investigation. From *in vitro* evidence to clinical evidence as monotherapy or as a sparing agent with chemotherapy and immunotherapy, each drug class (metformin, rosiglitazone, GLP-1RAs, SGLT2 inhibitors) presents unconventional mechanisms, benefits, and limitations that require elucidation (54). Key priorities include identifying which cancer types and patient subgroups are most likely to benefit from specific GLDs, determining optimal dosing and timing, and understanding potential drug-drug interactions with chemotherapy and immunotherapy agents.

Targeting shared pathways. The shared molecular pathways between diabetes and PDAC (PI3K/AKT/mTOR, NF-κB, YAP/TAZ) represent rational therapeutic targets. Future research should explore whether drugs targeting these pathways could simultaneously address both metabolic dysfunction and tumor progression.

### 6.2 Clinical research priorities

#### 6.2.1 Randomized controlled trials for NOD-based screening

The Early Detection Initiative (EDI). A landmark randomized controlled trial is currently underway to address the critical question of whether algorithm-based screening in patients with NOD can improve early detection of PDAC. The EDI uses a modified Zelen’s design with post-randomization consent, aiming to enrol 12,500 subjects. Subjects randomized to the Intervention arm undergo END-PAC risk stratification, with those scoring high undergoing CT imaging for PDAC detection. The study is designed to improve the detection of sporadic PDAC when surgical intervention is possible (55).

Priority research questions for future trials:

- Does NOD-based screening reduce PDAC-specific mortality?
- What is the optimal screening interval for high-risk NOD patients?
- What is the cost-effectiveness of different screening strategies in diverse healthcare systems?
- Can biomarker-based triage reduce unnecessary imaging in low-risk NOD patients?

#### 6.2.2 Validation and improvement of prediction models

Machine learning approaches. Further refinement and external validation of risk-stratification tools remain essential. An XGBoost machine learning model achieved an AUC of 0.80 (95% CI 0.76-0.85) compared to 0.63 and 0.68 for other contemporary models. Importantly, the XGBoost model was the only model that detected at least 50% of patients with cancer one year after the onset of diabetes (44).

A separate study using real-world clinical data from a Taiwanese EMR database (66,384 patients) developed a Linear Discriminant Analysis (LDA) model that generated an AUROC of 0.9073 for predicting pancreatic cancer risk in diabetic patients (43).

As effective targeted therapies such as KRAS pathway inhibitors become increasingly available for PDAC, the clinical value of early detection screening will be amplified. Retrospective analyses of pre-diagnosis metabolic and weight trajectories within prospective therapeutic trial cohorts should be considered, to link screening algorithm performance with access to novel curative intent therapies and patient survival outcomes.

Future directions:

- External validation of machine learning models in diverse populations
- Development of models incorporating genetic and biomarker data alongside clinical variables
- Prospective testing of model-guided screening algorithms
- Assessment of model performance across racial and ethnic groups

#### 6.2.3 Biomarker validation studies

Adrenomedullin as a screening tool. The Pancreatic Cancer in New-Onset Diabetes (PaCANOD) cohort study is prospectively enrolling 440 patients aged 45–75 years within 2 years of first fulfilling ADA criteria for diabetes. Patients are divided into groups based on high versus low adrenomedullin levels and will undergo 3 years’ follow-up to detect pancreatic cancer development. Identifying a marker for pancreatic cancer among high-risk patients such as new-onset diabetics might lead to the identification of a subpopulation needing to be screened (59).

CPDPC biomarker validation. The CPDPC has established a biobank of clinically annotated biospecimens and is conducting validation studies of promising biomarkers (56). Future research should prioritize:

- Phase 3 validation of the most promising biomarkers (including adrenomedullin, pancreatic polypeptide, S100A8/A9, and miRNA panels)
- Development of multi-marker panels for improved sensitivity and specificity
- Integration of biomarkers with clinical risk scores
- Assessment of biomarkers for monitoring treatment response and detecting recurrence

### 6.3 Clinical practice and guideline development

#### 6.3.1 Standardizing diagnostic criteria for type 3c diabetes

The need for consensus. Currently, there are no standardized diagnostic criteria for T3cDM globally, unlike those for T1DM and T2DM. The CPDPC has identified this as a major knowledge gap requiring urgent attention (56). A conceptual framework has been previously proposed (Ewald and Bretzel criteria), but empirical data are needed to develop validated diagnostic criteria (51).

Future priorities:

- Development of evidence-based diagnostic criteria for T3cDM using prospective cohort data
- Validation of diagnostic criteria in diverse populations
- Creation of clinical algorithms to distinguish T3cDM from T2DM in routine practice
- Incorporation of T3cDM diagnostic criteria into international diabetes guidelines

#### 6.3.2 Integrating diabetes and oncology care

Multidisciplinary approaches. Few clinicians specialize at the intersection of oncology and diabetology, and this disciplinary gap has limited progress in the field (39). Future initiatives should:

- Establish multidisciplinary clinics for patients with new-onset diabetes at high risk of PDAC
- Develop clinical pathways for coordinated diabetes and cancer care
- Create educational programs to increase awareness of T3cDM among primary care physicians, endocrinologists, and gastroenterologists
- Integrate pancreatic cancer risk assessment into routine diabetes management for older adults with new-onset diabetes

### 6.4 Emerging technologies and future innovations

#### 6.4.1 Artificial intelligence and precision medicine

The application of artificial intelligence to predict pancreatic cancer risk among diabetic patients represents a rapidly evolving field. Future research should focus on:

- Integration of multi-modal data (clinical, genomic, proteomic, imaging) into AI models
- Development of explainable AI models that provide interpretable risk predictions
- Prospective validation of AI models in clinical trial settings
- Assessment of AI-guided screening strategies for cost-effectiveness and clinical utility

#### 6.4.2 Liquid biopsy and early detection

Liquid biopsy approaches, including circulating tumor DNA (ctDNA), exosomes, and circulating tumor cells, offer promise for early PDAC detection in high-risk NOD patients. Future research priorities include:

- Validation of ctDNA-based tests (such as the cfDNA epigenomic blood test evaluated by Vilalta et al., 2026 (45)) in large prospective cohorts
- Integration of liquid biopsy with clinical risk scores
- Assessment of longitudinal biomarker changes to detect the transition from NOD to PDAC
- Development of multi-analyte panels incorporating exosomal markers

## 7 Conclusion

The bidirectional relationship between diabetes mellitus and PDAC represents one of the most clinically significant and mechanistically fascinating examples of cancer-host interaction. Over the past three decades, substantial progress has transformed this association from a mere epidemiological observation into a well-characterized biological and clinical paradigm with tangible implications for early detection, prevention, and patient management.

### 7.1 Summary of key findings

Multidimensional mechanisms underlie bidirectional crosstalk. Compelling epidemiological data support bidirectional causal links between T2DM and PDAC, with postsurgical glycemic remission confirming the paraneoplastic nature of PDAC-related T3cDM. Multiple interconnected molecular pathways mediate this reciprocal crosstalk, spanning metabolic signaling, tumor-secreted factors, fibrosis, inflammation and microbiome perturbations (2, 6, 37, 38).

Clinical applications are emerging but require validation. The recognition that approximately 0.8-1.0% of individuals aged ≥50 years with new-onset diabetes will develop PDAC within three years has established NOD as a critical window for early detection (44). The END-PAC and machine learning-based risk scores can stratify high-risk NOD patients (3, 44), and cost-effectiveness analyses suggest that risk-based screening approaches are economically viable when cancer incidence exceeds 2% (42). The EDI randomized controlled trial is currently evaluating whether algorithm-based screening in NOD patients improves early PDAC detection (55).

Antidiabetic drugs remain controversial. While observational studies have suggested that metformin may reduce PDAC risk and improve prognosis, umbrella reviews have demonstrated that the evidence is plagued by severe bias, immortal time bias, and low quality, with no high-quality evidence supporting metformin’s beneficial effects in PDAC (21). For GLP-1 receptor agonists, meta-analyses show no significant association with pancreatic cancer overall, though a significant increase was found in the subgroup with background medications (RR: 1.85, 95% CI 1.05-3.26, p=0.03) but not without background medications (49, 53).

Type 3c diabetes is substantially underdiagnosed. Widespread misclassification of T3cDM as classic T2DM continues to impede timely PDAC detection (7). This misdiagnosis leads to delayed pancreatic imaging, inappropriate treatment with insulin sensitizers, and failure to recognize and treat exocrine pancreatic insufficiency, which affects 80% of T3cDM patients (7, 51).

Consensus is urgently needed. The field has been fractured around inconsistent disease descriptions and a lack of standardized diagnostic criteria for T3cDM. As highlighted by Hill et al., “This disciplinary gap has limited visibility at major international conferences and hindered consensus on endpoints, cohorts, and study timing” (39).

### 7.2 Clinical and research implications

NOD-based screening represents the most feasible path to early detection. For the first time, a well-defined high-risk population-–individuals aged ≥50 years with new-onset diabetes-–has been identified in which the pre-test probability of PDAC (0.8-1.0% over three years) is sufficiently high to consider targeted screening. While current detection rates fall below cost-effectiveness thresholds, ongoing research, including the EDI trial and biomarker validation studies, may identify strategies to improve screening performance.

Drug repurposing remains promising but requires rigorous evaluation. The multifaceted actions of glucose-lowering drugs in cancer treatment warrant further investigation, with priority areas including identifying which patient subgroups are most likely to benefit, determining optimal dosing and timing, and understanding potential drug-drug interactions with chemotherapy and immunotherapy agents (54).

Shared genomic biomarkers offer diagnostic opportunities. Recent bioinformatics analyses have identified common differentially expressed genes (TOP2A, BIRC5, RRM2, ALB, MUC1, E2F7, and CEBPZ) as potential genomic biomarkers linking T2D and PDAC (57, 58). Validation of these biomarkers in prospective cohorts could lead to novel diagnostic approaches.

Despite substantial progress, several critical questions remain unanswered (**Table 6**).

**Table 6:** Key unresolved questions and future directions.

Question	Priority	Current Status
What are the specific tumor-derived factors that cause β-cell dysfunction?	High	Preclinical evidence established; human validation needed
Does NOD-based screening reduce PDAC-specific mortality?	High	EDI RCT ongoing (55)
What is the optimal risk stratification strategy for NOD patients?	High	Machine learning models promising; external validation needed
Can validated diagnostic criteria for T3cDM be developed?	High	CPDPC consensus development underway (56)
What is the true risk-benefit profile of GLP-1RAs for pancreatic cancer?	Medium	Meta-analyses show mixed results; longer-term studies needed
Can glucose-lowering drugs be repurposed for PDAC treatment?	Medium	Preclinical evidence; RCTs needed

### 7.4 Concluding remarks

The bidirectional relationship between diabetes and PDAC has evolved from a clinical curiosity to a paradigm of cancer-host interaction with direct translational implications. Long-standing T2DM is a modifiable risk factor for PDAC, while new-onset type 3c diabetes serves as an early specific warning signal of underlying PDAC. These two phenomena are interconnected through overlapping metabolic, inflammatory, and molecular pathways that create a self-reinforcing cycle accelerating both tumor progression and metabolic deterioration.

Clinically, NOD-based risk stratification represents the most feasible path to improving early PDAC detection. The ongoing EDI randomized controlled trial will provide definitive evidence regarding the efficacy of this approach. Meanwhile, the widespread misdiagnosis of T3cDM as T2DM represents an immediate opportunity for quality improvement: routine consideration of pancreatic pathology in older adults with new-onset diabetes who lack typical T2DM features (obesity, metabolic syndrome) could accelerate diagnosis and improve outcomes.

Future breakthroughs will likely emerge from multi-omics approaches that elucidate the precise molecular mediators of PDAC-induced diabetes, prospective validation of novel biomarkers, successful completion of the EDI trial, and consensus-driven development of diagnostic criteria for T3cDM. As the field matures, the integration of oncology and diabetology-–both in research and clinical practice-–will be essential to translate mechanistic insights into tangible improvements in patient outcomes.

The bidirectional relationship between diabetes and pancreatic cancer is no longer merely an association to be observed but a relationship to be leveraged for early detection, prevention, and treatment. The path forward requires continued collaboration between basic scientists, clinical researchers, and healthcare providers to close the disciplinary gap that has historically limited progress (39).
